# Developing a ‘moral compass tool’ based on moral case deliberations: A pragmatic hermeneutic approach to clinical ethics

**DOI:** 10.1111/bioe.12617

**Published:** 2019-07-24

**Authors:** Laura Hartman, Suzanne Metselaar, Guy Widdershoven, Bert Molewijk

**Affiliations:** ^1^ Department of Medical Humanities University of Amsterdam the Netherlands; ^2^ Centre for Medical Ethics, University of Oslo Norway

**Keywords:** clinical ethics support, ethics support tool, hermeneutics, methodology, moral case deliberation, pragmatism, theoretical reflection

## Abstract

Although moral case deliberation (MCD) is evaluated positively as a form of clinical ethics support (CES), it has limitations. To address these limitations our research objective was to develop a thematic CES tool. In order to assess the philosophical characteristics of a CES tool based on MCDs, we drew on hermeneutic ethics and pragmatism. We distinguished four core characteristics of a CES tool: (a) focusing on an actual situation that is experienced as morally challenging by the user; (b) stimulating moral inquiry into the moral concepts, questions and routines in the lived experience of the CES tool user; (c) stimulating moral learning by exploring other perspectives; and (d) incorporating contextual details. We provide an example of a CES tool developed for moral dilemmas over client autonomy. Our article ends with some reflections on the normativity of the CES tool, other application areas and the importance of evaluation studies of CES tools.

## 1 INTRODUCTION

A moral case deliberation (MCD) is a reflective dialogue, in which, through a structured conversation method, a concrete moral issue that has been experienced is analysed by a group of practitioners in order to come to a shared moral perspective and a deepened or new insight as to which values and norms should prevail in the situation.1Molewijk, A. C., Abma, T. A., Stolper, M., & Widdershoven, G. A. M. (2008). Teaching ethics in the clinic. The theory and practice of moral case deliberation. *Journal of Medical Ethics,* *34*(2),120–124. Molewijk, A. C., Verkerk, M., Milius, H., & Widdershoven, G. A. M. (2008). Implementing moral case deliberation in a psychiatric hospital: Process and outcome. *Medicine, Health Care, and Philosophy,* *11*(1), 43–56. Stolper, M., Molewijk, A. C., & Widdershoven, G. A. M. (2016). Bioethics education in clinical settings: Theory and practice of the dilemma method of moral case deliberation. *BMC Medical Ethics,* *17*(1), 45. This dialogue is moderated by a trained facilitator who stimulates joint reflection and dialogue and keeps a focus on the moral dimension and the central moral question.2Stolper, M., Molewijk, A. C., & Widdershoven, G. A. M. (2015). Learning by doing. Training health care professionals to become facilitator of moral case deliberation. *HEC Forum,* *27(*1), 47–59. MCD is an established practice of clinical ethics support (CES) in the Netherlands.3Dauwerse, L., Dauwerse, L., Stolper, M., Widdershoven, G. A. M., & Molewijk, A. C. (2014). Prevalence and characteristics of moral case deliberation in Dutch health care. *Medicine, Health Care, and Philosphy,* *17*(3), 365–375. It is also implemented in other countries in Europe, in which the terms ‘ethics case reflection’ and ‘ethics reflections group’ are also used, indicating similar kinds of CES meetings.4Svantesson, M., Anderzen‐Carlsson, A., Thorsen, H., Kallenberg, K., & Ahlstrom, G. (2008). Interprofessional ethics rounds concerning dialysis patients: Staff’s ethical reflections before and after rounds. *Journal of Medical Ethics,* *34*(5), 407–413. Svantesson, M., Lofmark, R., Thorsen, H., Kallenberg, K., & Ahlstrom, G. (2008). Learning a way through ethical problems: Swedish nurses’ and doctors’ experiences from one model of ethics rounds. *Journal of Medical Ethics,* *34*(5), 399–406. Lillemoen, L., & Pedersen, R. (2013). Ethical challenges and how to develop ethics support in primary health care. *Nursing Ethics*, *20*(1), 96–108. Lillemoen. L,, & Pedersen, R. (2015). Ethics reflection groups in community health services: An evaluation study. *BMC Medical Ethics*, *16*, 25. Gronlund, C. F., Dahlqvist, V., Zingmark, K., Sandlund, M., & Soderberg, A. (2016). Managing ethical difficulties in healthcare: Communicating in inter‐professional clinical ethics support sessions. *HEC Forum*, *28*(4), 321–338. Rasoal, D., Kihlgren, A., James, I., & Svantesson, M. (2016). What healthcare teams find ethically difficult. *Nursing Ethics,* *23*(8), 825–837. Bartholdson. C., Molewijk, B., Lützén, K., Blomgren, K., & Pergert, P. (2018). Ethics case reflection sessions. *Nursing Ethics*, *25*(2), 199–211. Although there are differences, the same key elements are present; a group of participants, a facilitator, a method to structure the conversation, one concretely experienced case and a moral question.5Rasoal. D., Skovdahl, K., Gifford, M., & Kihlgren, A. (2017). Clinical ethics support for healthcare personnel: An integrative literature review. *HEC Forum*, *29*(4), 313–346.


MCD is an example of an approach in CES that emphasizes the importance of practicing ethical reflection together with practitioners embedded in daily care practices and routines, instead of a more detached reflection on care practices. Furthermore, within MCD the experience and the moral expertise of care practitioners themselves are central, rather than theoretical knowledge of, for instance, an ethicist.6Abma, T.A., Baur, V.E., Molewijk, A. C., Widdershoven, G. A. M. (2010). Inter‐ethics: Towards an interactive and interdependent bioethics. *Bioethics*, *24*(5), 242–255. MCD takes experiences, moral questions and moral knowledge of care practitioners as the starting point for ethical reflections. Trained professionala who facilitate an MCD neither give advice with respect to the case at hand nor do they have to be an expert on the specific topic. Yet the MCD facilitator is an expert in facilitating a moral inquiry through a constructive dialogue, in recognizing and extracting the moral dimension in common team discussions, and in systematically reflecting upon how MCD participants reason and draw (different) normative conclusions based on specific facts of the case at hand.

Several evaluation studies have shown that participants report having positive experiences with MCD. MCD contributes to the joint understanding of the ethical issue at stake, improves decision‐making processes and multidisciplinary cooperation, supports the development of the moral competence of MCD participants and clarifies what quality of care entails.7Stolper et al., op. cit. note 2. Seekles, W., Widdershoven, G. A. M., Robben, P., Dalfsen, van, G., & Molewijk, A. C. (2016). Evaluation of moral case deliberation at the Dutch health care inspectorate: A pilot study. *BMC Medical Ethics*, *17*(1), 31. Janssens, R. M., Zadelhoff, van E., Loo, van G., Widdershoven, G. A. M., & Molewijk, A. C. (2015). Evaluation and perceived results of moral case deliberation: A mixed methods study. *Nursing Ethics*, *22*(8), 870–880. Weidema, F. C., Molewijk, A. C., Kamsteeg, F., & Widdershoven, G. A. M. (2015). Managers’ views on and experiences with moral case deliberation in nursing teams. *Journal of Nursing Management,* *23*(8), 1067–1075. Hem, M. H., Pedersen, R., Norvoll, R., & Molewijk, A. C.. (2015). Evaluating clinical ethics support in mental healthcare: A systematic literature review. *Nursing Ethics*, *22*(4), 452–466. Yet MCD also has some limitations. In essence, There should be these:

1. Lessons learned from individual MCDs are not shared in order to be used in other contexts.
2. MCD allows for only a limited number of people to join the reflection (about 8–12 per session). This allows relatively few professionals to learn and profit from the mutual learning process and insights generated by the joint reflection.
3. MCDs are relatively expensive and time consuming (sessions take on average 1 to 2 hr), have to be planned ahead and need a trained facilitator.8Stolper et al., op. cit. note 2. These characteristics require organizational effort and imply logistical challenges.
4. MCD deals with one individual concrete case. It does not address other, similar cases. If MCD is the only means of CES being used in an institution, there is a risk that the MCD sessions tackle the same kind of moral question over and over again whereas the systemic causes of these moral questions may not be recognized and addressed in a more general way.

In order to deal with these limitations, a large health care organization in the Netherlands where we successfully implemented MCD asked us to develop an additional, tailor‐made CES tool based on the insights gained in a series of MCDs that could be used more spontaneously and which could be distributed more easily through the whole organization. Hence, we were challenged to think about how to develop a *theme*‐based tailor‐made tool for this organization, inspired by the insights obtained by a series of MCDs on a specific topic. The goal of this CES tool was not to replace MCD but to provide CES *complementary to* MCD. Given the importance and widespread presence of moral dilemmas concerning client autonomy, the decision was made to focus on this issue. This objective raises important theoretical questions. Which characteristics should the CES tool possess? To what extent can we process lessons learned from earlier MCDs on client autonomy in this CES tool? What kind of results or outcomes should such a CES tool bring about? What kind of normative guidance is possible with the CES tool? In this article we seek to answer these questions.

In the following, we first describe some key theoretical notions of MCD derived from hermeneutics and pragmatism. These theoretical notions concern the concepts *moral knowledge* and *moral learning.* Then, we consider the implications of these notions for the development of a theme‐based CES tool based on case‐based MCDs. We describe the four core characteristics that a CES tool should have, based on this theoretical framework. Then we provide an example of a CES tool developed for moral dilemmas addressing the moral theme of client autonomy for a health care institution that provides long‐term care to a diverse range of clients (such as elderly care and mental health care).9We have described the development and content of this CES tool in more detail elsewhere. See Hartman, L. A., Metselaar, S., Molewijk, A. C., & Widdershoven, G. A. M. (2018). Developing a moral compass for dealing with dilemmas around autonomy in long‐term care: An ethics support tool based on a series of moral case deliberations. *BMC Medical Ethics*, *19*(1), 97. Our article ends with some reflections on the normativity of the CES tool, other application areas and the importance of evaluation research of CES tools.

## 2 THEORETICAL BACKGROUND OF MCD: EXPERIENCE AND JOINT LEARNING

We describe the theoretical background of MCD here in a general way. This means that we focus on two philosophical traditions, i.e. hermeneutics and pragmatism. Rather than going into detail on the different theories and philosophers that belong to these traditions, we focus on the core concepts, philosophical views and common characteristics of both traditions. We will select some key theoretical concepts that help us interpret and specify the proceedings and normative status of MCD as CES. This in turn allows us to formulate some implications for developing a theme‐based CES tool based on these MCDs (paragraph 3).

Furthermore, our use of the concepts ethics and morality needs elucidation. We use the concept morality to refer to our moral routines, consisting of a complex web of norms, values, obligations, etc. and reserve the term ethics for the actual discussion of or reflection on these norms, values and obligations.10Dewey J. (1922, 2002). *Human nature and conduct*. Mineola, NY: Dover. Keulartz, J., Schermer, M., Korthals, M., & Swierstra, T. (2004). Ethics in technological culture: A programmatic proposal for a pragmatist approach. *Science, Technolology & Human Values, 29*(1), 3–29. Different uses are morals as expressing community norms and ethics as guiding individual decisions or that ethics is about rules (as in ‘code of ethics’) and morality is about more complex situation‐sensitive judgement. According to this distinction, as soon as people start discussing, reflecting on or questioning their morality, they are engaged in ethics. So a conversation on an ethical issue by the coffee machine, a joint reflection within an MCD meeting, a discussion in an ethics committee or a commentary in an ethics journal are all examples of doing ethics (some obviously more structured then others). We understand ethics as the *activity of* discussing, researching, asking questions about, scrutinizing or reflecting on an existing morality, it entails *being engaged with* morality and asking questions about the right thing to do. This reflection on morality, departing from our experience and focusing on moral learning, is the core ingredient of MCD sessions.

### 2.1 Hermeneutics

According to philosophical hermeneutics, ethics is not merely a matter of theoretical insight, but starts with practical experience. We can only come to know what is morally right by dealing with ethical problems in practice, starting from experience.11Widdershoven, G. A. M., Molewijk, A. C., & Abma T. A. (2009). Improving care and ethics: A plea for interactive empirical ethics. *American Journal of Bioethics,* *9*(6–7), 99–101. Widdershoven, G. A. M., & Metselaar, S., (2012) Gadamer’s *Truth and Method* and moral case deliberation in clinical ethics. In Sneller, R., Kasten, M. J. A., & Paul, H. J., (Eds.), *Hermeneutics and the humanities: Dialogues with Hans‐Georg Gadamer* (pp. 287–305). Leiden, the Netherlands: Leiden University Press. How one perceives and interprets the world is dependent on one’s personal experience, personal history and the norms and values of the culture and family in which one grew up, but also the one's education and professional norms of the organization. Judgements, opinions and deliberations about what is right cannot be made from a neutral, birds eye point of view, but always start from a ‘specific here’ with all its contextually bound knowledge and information.12Verkerk, M., & Lindemann, H. (2012). Toward a naturalized clinical ethics. *Kennedy Institute of Ethics Journal*, *22*(4), 289–306. This means that an ethical question or ethical dilemma is inherently related to the concrete experiences of people involved (i.e. an ethical question or dilemma does not exist independently).

Our embeddedness in the world, according to hermeneutics, implies a specific view on both moral knowledge and moral learning. We do not possess moral knowledge in an abstract and general way and then apply this moral knowledge to concrete circumstances. Instead, the understanding and interpretation of our moral concepts is created *within the situated contexts of concrete practices.* This understanding is also influenced by the historical and cultural context of the knower*.* So moral concepts like autonomy and dignity acquire meaning *through their use* within practices over time. What is right always has to be explored in the practice in which the ethical issue arises.13Gadamer, H.G. (1975). *Truth and method*. New York, NY: Seabury Press. This is essentially what is done in an MCD; the meaning and understanding of the relevant moral concepts is explored, shared and contrasted by a group of practitioners through a dialogue on a concrete experience of an ethical dilemma in a practice, involving an exchange of views which are directly related to personal experience.14Widdershoven, G. A. M., & Metselaar, S., op. cit. note 11. Widdershoven, G. A. M., & Molewijk, A. C. (2010). Philosophical foundations of clinical ethics: A hermeneutic perspective. In Schildmann, J., Gordon, J. S., & Vollmann, J. (Eds.), *Clinical ethics consultation theories and methods, implementation, evaluation* (pp. 37–51). Farnham, UK: Ashgate. Moreover, *which moral concepts are relevant* is also explored and discussed by the participants based on their moral experience and not defined beforehand.

In line with this interpretation of *moral knowledge, moral learning* also gets a specific interpretation. Moral learning starts from a confrontation between our experience and our surroundings. We continuously reflect on, learn from and alter our judgements on the basis of our encounters with the world and with others. The knower and the known are interrelated, and the background of the knower influences what is learned from a specific situation. The complex background that one has acquired by moving through the world influences what one observes and learns from a new situation.15Ohnsorge, K. (2015). Investigations in hermeneutic bioethics (PhD Thesis). Free University, Amsterdam, the Netherlands.. This implies that all observers of *one situation* may learn something different, depending on their specific background by the confrontation with other perspectives. This entails an embodied, social and dynamic theory of moral learning, for which engaging with and being exposed to other practices and viewpoints is essential. According to hermeneutics, moral learning is neither the act of leaving one’s views fully behind, nor of reaching a definite, objective view of the situation. The idea is that one understands one’s own position as limited, not as totally wrong. Further exposure to new perspectives may lead to new knowledge, but this process does not result in objective knowledge. There is always another perspective, and human learning is always finite. However, this does not exclude the role of ethical theory and critical thinking in hermeneutics. Ethical theory can be of great importance for moral deliberation in specific contexts.

Following this approach, an MCD session can be regarded as a place where moral learning is actively encouraged. The participants jointly explore and reflect upon a concrete ethical question from their daily practice and enter in a dialogue, questioning each other’s normative convictions, viewpoints and presuppositions.16Widdershoven, G. A. M., & Molewijk, A. C., op. cit. note 14. MCD stimulates reflecting on and scrutinizing one’s morality and the norms and values one holds, making them explicit and subject to change by the interaction with the morality, meaning and specific interpretations of values and norms of the other participants (inspired by *their* own contextual backgrounds and personal history). During MCD, the participants are stimulated to ask questions about each other’s views (instead of trying to convince each other and trying to defend their own point of view). Listening and exploring various points of view is crucial in MCD, and is also characterized as the Socratic attitude.17Kessels, J. (1997). Socrates op de Markt: Filosofie in Bedrijf. Amsterdam, the Netherlands: Boom. Korthagen, F. A. J., & Kessels, J. P. A. M. (1999). Linking theory and practice: Changing the pedagogy of teacher education. *Educational Researcher*, *28*(4), 4–17. Abma, T. A., Molewijk, A. C., & Widdershoven, G. A. M. (2009). Good care in ongoing dialogue. Improving the quality of care through moral deliberation and responsive evaluation. *Health Care Analysis*, *17*(3), 217–235. Rudnick, A. (2002). The ground of dialogical bioethics. *Health Care Analysis*, *10*(4), 3912402.


The Socratic attitude implies that moral learning requires perceptiveness and sincere openness. A person should be open to seeing a concrete situation from various perspectives. Contextual details of the situation are morally significant. Moral learning takes place when a person actively reflects on (the limits of) their own morality and their own interpretation of the situation and takes other viewpoints and experiences of the situation seriously into account. Moral learning, in sum, implies developing a richer and more nuanced understanding of the situation. This new understanding is not just cognitive, but also embodied within actions – by moving through the world, one learns. Moral learning enables a person to see a situation differently and to act on newly acquired insights.

### 2.2 Pragmatism

Like hermeneutics, pragmatism emphasizes the importance of *concrete practices* for our understanding of moral concepts.18McGee, G. (2003). *Pragmatic bioethics*. Cambridge, MA: MIT Press. Instead of searching for an objective or universal moral truth, pragmatism understands our moral concepts as *tools* that acquire their specific meaning within dynamic contexts and practices. The concept of tools emphasizes the instrumental nature of our concepts. Correspondingly, our moral concepts should be evaluated on their use and usefulness in practices (i.e. do they solve the problems they were designed to solve? Do they help making sense of a situation? Do they enable successful responses to new problems?).19Anderson E. (2014). Dewey’s moral philosophy. *Stanford Encyclopedia of Philosophy Archive*. Retrieved from https://plato.stanford.edu/archives/spr2014/entries/dewey-moral
 According to pragmatism, the meaning of moral concepts changes in response to the situations in which they are applied.

According to pragmatism, our morality is embedded in our actions and our self‐understanding. This is mostly so self‐evident that we are hardly aware of it. The values and norms that one acquires by growing up and learning the norms of, for instance, a profession are most of the time not explicit, but implicitly play a role in the way the professional approaches a situation. We take these implicit values and norms for granted as they are embedded in our (moral) routines, habits, actions, interactions and intuitive moral judgements. The background that is acquired by moving through the world influences what is considered to be the right thing to do; it is one’s implicit point of reference.20Swierstra, T., & Rip, A. (2007). Nano‐ethics as NEST‐ethics: Patterns of moral argumentation about new and emerging science and technology. *NanoEthics*, *1*(1), 3–20. Williams, B. A. O. (1985). *Ethics and the limits of philosophy*. Harvard, MA: Harvard University Press. We often live and enact our norms and values instead of reasoning about them in a cognitive way. Our morality shows in our behaviour.

Our moral routines or habits can, however, be challenged in certain situations: for instance when two values are competing in that situation, when one is confronted with another perspective on a situation or when one cannot take one’s moral routines for granted any more in the context of a new practice or technology.21Swierstra, & Rip, op. cit. note 20. At such moments, norms and values may become subject of reflection, debate and inquiry. Moral inquiry often starts when a person experiences a problem *with a practice*, when something needs to be done and one doubts the right course of action. But moral routines can also be challenged by an outsider perspective or an, up until that point, silent voice, like a patient’s perspective or the perspective of a minority group. According to the pragmatism of Dewey, moral inquiry is problem oriented instead of theoretically oriented.22Gouinlock, J. (1994). *The moral writings of John Dewey*. Amherst, NY: Prometheus. When we experience a moral dilemma our moral routines are not self‐evident any more.

MCD can stimulate a systematic moral inquiry in order to scrutinize our moral routines.23Inguaggiato, G., Metselaar, S., Porz, R., & Widdershoven, G. A. M. (2019). A pragmatist approach to clinical ethics support: Overcoming the perils of ethical pluralism. *Medine, Health Care, and Philosophy*. doi.org/10.1007/s11019‐018‐09882‐3 In MCD, presuppositions and moral routines are critically reflected upon within the practical situation at hand. In the end, this moral inquiry may lead to new insights about what is morally right, which again may lead to new moral routines. Take, for example, the implicit view on collegiality of a person. If the person holds the implicit norm that collegiality entails ‘always being loyal towards each other’, the behaviour of colleagues will be judged by this norm. This may give rise to a dilemma, for instance, when a colleague does something that seems to be wrong, and is disrespectful towards her clients. Should one be loyal towards this colleague and keep silent, or should one take action and try to change the situation? And in case of the latter; what action would be the right one on the situation? Should one confront the colleague or report her to the manager, or intervene when the specific event takes place?

Establishing the specific moral dilemma that this situation entails is personal: for other individuals this example may not even be a moral dilemma. Reflecting upon this situation in an MCD encourages one to make explicit one’s implicit norms (of which one may not even have been aware of until then). Within MCD, by means of a joint moral inquiry, participants will encounter other norms and values, and other interpretations of the facts of this situation. For instance, another participant may hold the implicit norm that true collegiality entails ‘being critical and truthful towards each other’. When different norms and values become explicit, participants are able to see more in a situation, reflect upon different norms (not just about collegiality, but maybe also about good care etc.). This might result in moral learning: making implicit moral routines explicit, scrutinizing these routines in a joint process of reflection and in the end come to new moral understandings and routines.

To conclude, both hermeneutics and pragmatism emphasize the dynamic and contextualized nature of morality. Both theories are oriented towards actions and put an emphasis on the importance of practices and contextual details to determine what is morally right. Moral knowledge is embedded in our actions and self‐understanding and moral learning takes place when one’s moral routines are challenged by another perspective. In the following we describe the implications of these theoretical reflections for developing a theme‐based CES tool for a series of case‐based MCDs.

## 3 IMPLICATIONS FOR DEVELOPING A THEME‐BASED CES TOOL

Based on the theoretical notions described above, we will now articulate four core characteristics the CES tool should have.

### 3.1 Characteristic 1: An problem that has been experienced as a starting point

As described above, practitioners start doing ethics when they experience a problem with their moral routines in a concrete situation, i.e. moral inquiry is problem oriented. As a result of a specific experience (that can be anything from a confrontation with the perspective of another person, critical questions from an outside perspective, a new technology or reading a book), one’s moral routines do not function as self‐evidently as they used to, which is when people often start experiencing moral doubts. At that moment they might experience the need to reflect on their morality. This is the moment in which practitioners might feel in need of CES, which is why the proposed CES tool starts with relating to the concrete issue that is experienced as morally troublesome, rather than with presenting abstract moral knowledge, general ethical issues or a predefined ethical dilemma that is not experienced based.

### 3.2 Characteristic 2: A focus on moral inquiry into the moral concepts, questions and routines within the lived experience of the CES tool user

Secondly, hermeneutics and pragmatism both emphasize that an ethical dilemma does not emerge from the facts but from a person’s experience of the facts. This experience differs according to the person and perspective or the position within the situation (for instance, related to the profession of the practitioner). Where one person may experience great difficulties, the other may not. The CES tool should therefore not define the ethical question beforehand but ask the user to articulate the ethical question for themselves.

Moral concepts acquire their meaning within the concrete care practices and can be different from person to person. For instance, collegiality may predominantly entail ‘loyalty’ to one person and ‘being critical’ to another person. According to this theoretical framework, moral concepts cease to have a steady fixed meaning that may be transferred to practitioners (for instance, in a textbook or argumentation card): instead their meaning has to be created by practitioners within their own actions, conversations and practices.

Thus, the CES tool does not start with a definition of, for instance, a concept like autonomy and neither does it search for a definition of autonomy but stimulates reflection on what a concept like autonomy means for the user of the CES tool with regard to their own ethical dilemma and practice.

### 3.3 Characteristic 3: A focus on moral learning by exploring other perspectives

Moral learning is understood here, in line with hermeneutics and pragmatism, as becoming aware of one’s (often implicit) moral routines, prejudices and assumptions, and broadening one’s moral perspective in dialogical confrontation with the perspectives of others.

To stimulate reflection on implicit moral assumptions, the CES tool should encourage the user to engage with the perspectives of other stakeholders in the situation at hand. Therefore, the CES tool should challenge the user to look at the situation from different perspectives; for instance, from the point of view of a client/patient, family member or other professional. Rules and regulations, or other normative frameworks can also be interpreted as another perspective. By venturing into what could be of moral importance for these other stakeholders (in terms of values, norms and principles) by trying to put oneself in the shoes of the other, moral learning in terms of becoming aware of one’s own perspective and broadening one’s moral horizon is encouraged.

### 3.4 Characteristic 4: Incorporating contextual details

According to both hermeneutics and pragmatism, what is right can only be determined *within a concrete situation*, incorporating all morally relevant contextual details. This includes the facts, norms and values of the different stakeholders in the situation at hand, but also the relevant rules and regulations that apply to the situation. This means that the CES tool should include contextual details, formulated in the terminology of health care professionals. This requires empirical research, elaborating, for instance, the facilities that are available in a health care organization, the strategies that are often mentioned to handle a moral dilemma etc. This information needs to be reinterpreted by the user and taken into account for the moral consideration of a specific issue.

## 4 CASE EXAMPLE: DEVELOPING A MORAL COMPASS AS A THEMATIC CES TOOL

To illustrate the four characteristics of a CES tool we describe the actual development of a theme‐based CES tool based on a series of MCDs. We refer to the CES tool as a moral compass, because through the learning process the users of this tool themselves develop moral guidance in the specific situation at hand. We use the term moral compass as a tool or instrument that provides CES. With the term moral compass we do not refer to an inner, psychological moral framework, as is done in other literature.24See for instance Thompson, L. J., (2010). The global moral compass for business leaders. *Journal of Business Ethics*, *93*(Suppl 1), 15–32. The specific context in which we developed the tool was a large health care organization in and around Amsterdam. This organization provides long‐term care for elderly people, for people with a mental disability and for psychiatric patients (both ambulatory and in assisted living facilities).

Our research objective was to develop a low threshold CES tool based on a series of MCDs on client autonomy in long‐term care, to be complementary to MCD. The aim of the CES tool is to make both the process and the content of a series of MCDs on client autonomy useful, more accessible to a wider group and less time‐consuming than a MCD session, thus addressing the limitations of MCD described above. Preliminary research showed that health care professionals in the organization encounter ethical dilemmas related to respecting client autonomy at the one hand and actively intervening for the sake of client’s well‐being in a situation at the other hand. Whereas the institution aims to provide demand‐driven care, professionals experience moral doubts, especially when they feel that the well‐being of clients would benefit more from active intervention. Dilemmas included, for instance, whether clients in an assisted living facility should be allowed to eat and drink whatever and whenever they wanted (even when they were at risk, for instance, because of diabetes), whether a client’s wish to live independently should be honoured (even when it was doubtful whether they were up to this), and whether clients should be pressed to quit drinking and smoking (even if they had few other joys in life). Because these kinds of issues were omnipresent in the organization we decided to focus the moral compass on dealing with dilemmas related to this particular theme.

We used a qualitative research design in which we analysed both the process and content of a series of MCDs, combined with reflections on the theoretical background of MCD. For the MCDs we used the dilemma method.25Stolper et al. op. cit. note 1. In total, 28 MCDs (10 transcripts and 18 summary reports) were analysed by means of a thematic content analysis. In various rounds, the results of the analysis were combined with theoretical reflections on CES and moral learning. The analysis of the 28 MCDs resulted in extra process questions and concrete content related to client autonomy in the draft of the moral compass (for example, by enumerating possible values and norms that might play a role in dilemmas over client autonomy). Consequently, the tool was developed and evaluated in three focus groups with health care professionals and adjusted based on their feedback. We have described the actual development of the tool, the empirical analysis of the MCDs and the content and structure of the CES tool in more detail elsewhere.26Hartman, et al. op. cit. note 9. In the following, we describe how the moral compass embodies the four characteristics distinguished above. We describe the CES tool according to the characteristics and chronologically (from question 1 to 6).

### 4.1 What is your dilemma? An problem that has been experienced as the starting point (characteristic 1)

According to pragmatism, moral inquiry is problem‐oriented. This means that people start reflecting on their morality when they encounter a problem or challenge with their existing moral routines. The moral compass takes an actual moral dilemma that has been experienced as its starting point and stimulates reflection on that experience, instead of offering general moral arguments beforehand. Therefore, the first question that is presented to the user is: ‘Q1 Sometimes, it is not self‐evident what the right thing to do is. What is your dilemma?’ (see Table 1). The title page is designed to sensitize possible users to the kind of problems the moral compass may be used for. To accompany the moral compass, we developed a poster to hang in common areas of care practitioners, with all kind of examples of issues (based on our analysis of the MCDs) for which the moral compass may be used.

**Table 1:** Six questions from the moral compass with explanations (translated from Dutch)

question	Questions and their explanations from the moral compass
q1	*Sometimes, it is not self‐evident what is the right thing to do.* What is your dilemma?
q2	*It may be helpful to clarify what exactly makes the situation difficult*. What causes you to have moral doubts in this situation?
q3	*By placing yourself into the shoes of others, you acquire valuable insight into different perspectives on the problem and on the right thing to do. With each question, ask yourself what would be important to different people, both in the long and short run.* What is important to whom? Q3A: Place yourself in the shoes of the client: what is important for the client? Q3B: What is important for you? Q3C: Place yourself in the shoes of the others who are involved in the situation; what is important for them (colleagues, network, other clients, management)? Q3D: What do rules and regulations say about this situation?
q4	*Think about what is important for all the parties involved (as answered for Q3). Please give a reply* *in which you take all perspectives into account.* Q4A What is most valuable to you in this situation? Q4B: Which actions go along with this?
q5	*Often, there is no perfect solution for a dilemma. Each choice has its disadvantages, because you simply cannot do everything that is important. It helps to be conscious of this, so you can possibly limit these disadvantages.* Q5A: What is a (possible) disadvantage related to your chosen course of action? Q5B: Can you do anything to compensate for this disadvantage?
q6	Are you able to deal with your dilemma after using this compass?

The user of the moral compass is asked to apply a series of questions to *their own moral dilemma at* *hand*, stimulating reflection and moral learning within the care practice of the user. The six questions are inspired by the dilemma method,27Stolper, et al. op. cit. note 1. but we also added some questions based on the qualitative analysis of the MCDs that are not mentioned in the dilemma method. For instance, we asked for values over both the long term and the short term of patient care, as the analysis of the MCDs showed that this was a particularly insightful question. Moreover, based on the qualitative analysis, we inserted a question asking explicitly what the institutional and professional rules and guidelines say about the case (which is not explicitly addressed with regular steps in the dilemma method) (see Q3).

### 4.2 What causes your moral doubts? A focus on moral inquiry into the moral concepts, questions and routines within the lived experience of the CES tool user (characteristic 2)

The CES tool should and cannot determine beforehand whether or not an ethical dilemma as such exists but asks the user of the tool to formulate her dilemma on the basis of her experience. From a hermeneutic perspective, ethical dilemmas do not exist independently but always within someone’s experience. The moral compass is aimed at helping the user understand what the ethical question is for the user herself. Sometimes people experience a sense of unease but need support in formulating what exactly the moral question entails. The second question of the moral compass is intended to establis what it is about the situation that makes it so difficult and what the moral dilemma entails for the user: ‘It may be helpful to clarify what exactly makes the situation difficult. What causes you to have moral doubts in this situation?*’* (see Table 1). This question is illustrated with several examples of ethical dilemmas over patient autonomy, such as; ‘Who am I to tell the client what is good for him?’, ‘Where does my responsibility for the client stop?’, ‘To what extent should I take into account the wishes and interests of the family?’ and ‘As daily caretaker, do I stand above or beside the client?’.

The moral compass invites the user to investigate which moral concepts are relevant for the user in their specific situation. A large variety of values that were mentioned in the MCDs studied are incorporated in the moral compass as possible examples and the users are asked which values are important for them in this situation. These are values which were often mentioned during the MCDs to deepen the moral understanding of a moral dilemma over client autonomy, for instance ‘well‐being’, ‘safety’, ‘health’, ‘freedom to make your own mistakes’, etc. These values and moral concepts are used for heuristic purposes: they are suggestions and need to be used only when they adequately resonate with the user’s experience and thoughts. So, in line with the theoretical framework above, this enables the users to reflect on those values that have a meaning for that specific situation, for a specific user (for another user, a different set of values could be relevant).

Question 4 is formulated as follows: ‘Think about what is important for all the parties involved. Please make a consideration in which you take all perspectives into account’*.* Inspired by hermeneutics, the user is asked to take all the perspectives into account, before being asked (Q4A): ‘What is most valuable to you in this situation?’ (Q4B): ‘Which actions go along with this?’ Secondly, inspired by the tragic character of a dilemma, Q5 explains; ‘Often, there is no perfect solution for a dilemma. Each choice has its disadvantages, because you simply cannot do everything that is important. It helps to be conscious of this, so you can possibly limit these disadvantages’. (Q5A): ‘What is a (possible) disadvantage related to your chosen course of action?’ (Q5B): ‘Can you do anything to compensate for this disadvantage?’ These questions encourage the user to find a way out of their own experienced ethical dilemma, *after *they also looked at the dilemma from different perspectives (see characteristic 3).

### 4.3 What is important to whom? A focus on moral learning by exploring other perspectives (characteristic 3)

In the moral compass, we use two ways to stimulate moral leaning, in line with the theoretical framework described above. Firstly, the moral compass stimulates looking at the moral dilemma experienced from different perspectives, as emphasized by hermeneutics. Question 3 reads: ‘By placing yourself into the shoes of others, you acquire valuable insight into different perspectives on the problem and on the right thing to do. With each question, ask yourself what would be important to someone, both in the long and short run. What is important to whom?’

The user is asked to place herself in the shoes of the patient/client and think about the values that could be important for the client in the specific situation at hand. Q3A asks: ‘Place yourself in the shoes of the client: what is important for the client?’ Also, the user is asked to think about what would or could be important for other stakeholders in the situation (including herself) in Q3B: ‘What is important for you?’ and Q3C: ‘Place yourself in the shoes of the others who are involved in the situation; what is important for them? (colleagues, network, other clients, management?)’. Finally, Q3D asks: ‘What do rules and regulations say about this situation?’, as these also constitute a perspective.

Secondly, we incorporated substantive content mentioned in the MCDs. Thus, the user is exposed to the other viewpoints, values, action strategies that were discussed in the 28 MCDs. This may also stimulate reflection on their own moral routines, and thereby stimulate moral learning. Examples are; ‘involve other parties or other experts (like an experiential expert or a crisis manager)’, ‘let the client make her own mistakes’, ‘consult with the family or team manager, or report the situation’.

### 4.4 Incorporating contextual details (characteristic 4)

Based on the qualitative analysis of the MCDs, we incorporated specific contextual details in the moral compass, for instance, by mentioning how care practitioners phrase certain moral issues, providing the user with examples of values that were often mentioned during the MCDs and giving an overview of action strategies suggested by care practitioners in the health care organization, as both pragmatism and hermeneutics emphasize the importance of contextual details for ethical reflection. For each question in the CES tool we incorperated suggestions, examples and phrases to inspire the CES tool user. For instance, a strategy to involve a colleague was often mentioned during the MCDs as a way out of the moral dilemma. In this way, the care giver who experienced the moral dilemma can maintain a relationship of trust with the client, while another caregiver tries to intervene in the situation. We incorporated this strategy in the moral compass as a possible strategy: ‘leaving the intervention to another colleague, to maintain the relationship of trust between the caregiver and the client’.

Another example based on the analysis of the MCDs is that the participants of the MCD often do not choose between either intervening on the one hand or respecting the uttered wishes of the client on the other hand. Often, they try to find a middle ground by initiating a respectful and ongoing dialogue with the clients about the consequences of their uttered wishes (for instance, eating sweets when a client is diabetic). Thus, the participants of the MCDs try to respect the autonomy of the clients by treating them as equal partners in the conversation and maintaining a relationship of trust while at the same time taking responsibility for addressing the possible risks and harm of the uttered wishes: ‘ínvolve the client in decision‐making and jointly work towards a compromise’.

The qualitative analysis showed that many moral questions surrounding the theme of autonomy were complicated by the role of the family or by feeling that they were solely responsible for the situation. We also incorporated these aspects in the CES tool because they might be relevant for the user and the dilemma at hand. Finally, the moral compass addresses the question whether the user feels the dilemma has now been sufficiently dealt with. The final question (Q6) reads: ‘Are you able to deal with your dilemma after using this compass?’ If this is not the case, some follow‐up strategies are suggested, such as talking to your team manager or requesting an MCD. These are options that are available in that particular health care organization (in another health care organization, the follow‐up options would be different). The recommendation for an MCD also stresses the complementarity of the moral compass and MCD (see Table 1).

## 5 DISCUSSION

We have argued that a CES tool based on MCDs has to have certain characteristics in order to be in line with the theoretical CES approach of MCD. We identified four core characteristics: (a) a moral problem that has been experienced as starting point, (b) a focus on moral inquiry into the moral concepts, questions and routines within the lived experience of the CES tool user; (c) a focus on moral learning by exploring other perspectives; and (d) incorporating contextual details. These characteristics are formulated for a CES tool based on MCD and inspired by hermeneutics and pragmatics and do not necessarily apply in other kinds of CES.

By developing this CES tool we adressed three limitations of MCD. The tool combines insights from a series of 28 individual MCDs (addressing limitation 1). It is more easily accessible than MCD and can be distributed for the whole organization, (particularly when the CES tool is also digitally available, which the moral compass is, addressing limitation 2). Finally, it can be used without a trained facilitator present and takes less time than an MCD (addressing limitation 3). Limitation 4 is not addressed in the tool. The CES tool does not address systemic causes of frequently encountered moral dilemmas in practice on an organizational or policy level. This CES tool hads not been developed to address these levels, and another CES mechanism is needed to address limitation 4. However, the use of the moral compass could be used in acquiring information, trends and insights from the work floor in order to become helpful in addressing these possible causes. This requires further empirical research.

With the development of a thematic CES tool based on a series of MCDs we hve added to the variety of CES services that may be offered. Depending on the specific needs of a health care organization, CES tools have to be adjusted to the type of care (hospital care/long‐term care), the typical relationship with the patient/client in that specific type of care, the type of moral problems that care givers often experience, the general level of education of the users of the CES tools, the vocabulary that is often used, connected to actual practices and experiences of care practitioners, and the (action) strategies and facilities that are available in the specific care context. The question in which way the current ethics support tool can be used and adjusted in other contexts requires new experiences and further empirical research.

The CES tool that we have developed does not provide concrete normative guidance in the sense of rules that prescribe the right thing to do. Yet it is normative in a process‐oriented way, focusing on critical reflection and including different perspectives, values and action strategies that the participants of the MCDs found particularly helpful. It aims to stimulate moral learning by challenging the user to look at the moral issue from different angles and exposing the user to content from other perspectives that may be relevant to the situation. The tool does not entail an actual dialogue, as MCD does. Yet, in line with hermeneutic ethics, the tool aims at broadening one’s horizon by acquiring insights into the perspectives of others. In MCD an important step is to reconstruct the views of others. This idea is also used in the ethics support tool, which is based on the idea that making the effort to look at the case from different perspectives and think through the relevant norms and values will stimulate moral learning. There are multiple ways to stimulate moral learning; through an actual dialogue with people who think differently (as with an MCD), through individual use of this CES tool, but also by watching a movie or reading a text or going to the theatre. For future research in ethics support, we suggest further reflection upon and studying the differences in moral learning when using different approaches in ethics support. Finally, the CES tool provides the user with action strategies, contextual details of that health care organization and values and norms mentioned within MCDs, that may support the user with their own moral dilemma.

One strength of this study is the explicit reflection on the theoretical background of MCD for the development of this CES tool and the theoretical fit between the CES tool and MCD (independent of whether one agrees with this theoretical background). Other scholars have argued that theoretical reflection and consistency is often missing in the development of CES and CES‐related tools.28Reiter‐Theil, S., Mertz, M., Schurmann, J., Stingelin Giles, N., & Meyer‐Zehnder, B. (2011). Evidence – competence – discourse: The theoretical framework of the multi‐centre clinical ethics support project METAP. *Bioethics*, *25*(7), 403–412. Salloch, S., Schildmann, J., & Vollmann, J. (2012). Empirical research in medical ethics: how conceptual accounts on normative‐empirical collaboration may improve research practice. *BMC Medical Ethics,* *13*, 5. Schildmann, J., Molewijk, A. C., Benaroyo, L., Forde, R., & Neitzke, G. (2013). Evaluation of clinical ethics support services and its normativity. *Journal of Medical Ethics*, *39*(11), 681–685. Theoretical and normative viewpoints on CES are always implicitly present in CES and its evaluation.29Salloch, S., Wascher, S., Vollmann, J., & Schildmann, J. (2015). The normative background of empirical‐ethical research: First steps towards a transparent and reasoned approach in the selection of an ethical theory. *BMC Medical Ethics*, *16,* 20. In this study, we have been explicit about our theoretical viewpoints and made them part of the development process right from the start. In this way, theoretical reflection can influence the developmental process of the CES tool and steer the qualitative research process. For instance, in the qualitative analysis of the MCDs, we did not look for final answers or consensus, but focused on ingredients for heuristic purposes (e.g. offering examples of perspectives, arguments and values and norms), that were particularly insightful for the participants of the MCD.

In line with the pragmatic and hermeneutic approach we described above, it is now time to investigate whether the tool actually provides support in practice and in which way. It needs to be studied whether, and if so, under what conditions moral learning does actually take place in using the CES tool. The actual use of the CES tool in the care practices and by the stakeholders has to be evaluated. For example, how do we prevent users from simply picking certain already mentioned values and norms without really connecting with them? Is incorporating different perspectives enough to stimulate moral learning for the individual user of the CES tool and to what extent does this replace the actual presence of participants with other viewpoints in an MCD? Can such a CES tool be used individually or should it always be used in a small group to structure the dialogue? This evaluation research needs to be in accordance with the results and outcomes that we aim to bring about with the CES tool. The tool should, for instance, not be evaluated by investigating whether the use of the compass made a certain decision more easy or certain for the user, but whether moral learning has taken place. Evaluation of this kind of new and innovative CES tool is essential for the further development of CES and for assessing its actual contribution to health care practice.

## 6 CONCLUSION

In this article we discussed the theoretical framework for developing a theme‐based, tailor‐made CES tool on the basis of a series of MCDs. By reflecting on the theories of hermeneutics and pragmatism, we identified four core characteristics of a theme‐based CES tool based on MCDs: (a) a moral problem that was experienced as starting point; (b) a focus on moral inquiry into the moral concepts, questions and routines within the lived experience of the CES tool user; (c) a focus on moral learning by exploring other perspectives; and (d) incorporating contextual details. We illustrated these four core characteristics with a CES tool that we developed on client autonomy for a long‐term care organization. The identified characteristics and the way in which they were operationalized in the concrete tool may be helpful in developing other CES tools focusing on other themes in various health care organizations. Future evaluation studies should reflect upon the usefulness of this CES tool, the moral learning it evokes and its difference from moral learning within an MCD.

